# The influence of anti-cancer therapies on lymphocyte subpopulations of lung cancer patients

**DOI:** 10.3389/fimmu.2023.1239097

**Published:** 2023-08-28

**Authors:** Philipp Gessner, Belay Tessema, Markus Scholz, Ulrich Sack, Andreas Boldt, Andreas Kühnapfel, Christian Gessner

**Affiliations:** ^1^ Institute of Clinical Immunology, Faculty of Medicine, University of Leipzig, Leipzig, Germany; ^2^ Department of Respiratory Medicine, University Hospital Leipzig, Leipzig, Germany; ^3^ Department of Medical Microbiology, College of Medicine and Health Sciences, University of Gondar, Gondar, Ethiopia; ^4^ Institute for Medical Informatics, Statistics and Epidemiology (IMISE), University of Leipzig, Leipzig, Germany; ^5^ Pulmonary Practice, Leipzig, Germany

**Keywords:** non-small cell lung cancer, B cells, T cells, NK cells, anti-cancer therapies

## Abstract

**Introduction:**

There are limited data on the influence of different anti-cancer therapies on lymphocyte subpopulations and their relationships to survival of non-small cell lung cancer (NSCLC) patients. This study aimed to assess the effect of immunotherapy, chemotherapy, immunochemotherapy, adjuvant chemotherapy after surgery, and antibodies against Vascular Endothelial Growth Factors (VEGF) on B cell, T cell, and NK cell subpopulations, and the survival time of NSCLC patients.

**Methods:**

A total of 32 consecutive NSCLC patients were recruited at Pulmonology Clinic, Leipzig from January 2018 to March 2020 and enrolled in this study. Immunophenotyping was done using a FACS Canto II flow cytometer (BD Biosciences) before the administration of the planned therapy and during therapy with up to 7 observational windows for each patient targeting 130 immunologic parameters.

**Results:**

Absolute transitional B cells was significantly increased after immunotherapy (p = 0.032), immunochemotherapy (p = 0.030), and antibodies against VEGF (p = 0.024). Similarly, absolute counts and percentage of B cells were significantly increased after adjuvant chemotherapy (p = 0.023). However, absolute counts and percentage of transitional B cells are significantly decreased after chemotherapy (p = 0.001). Activated cytotoxic T cells were significantly increased after immunotherapy (p = 0.031) and immunochemotherapy (p = 0.030). The overall survival rate of NSCLC patients was 31%.

**Conclusions:**

In conclusion, this study suggests that different types of anti-cancer therapies affect lymphocyte subpopulations of NSCLC patients. Further large-scale and multicentre studies are required to confirm our results and to evaluate the prognostic value of lymphocyte subpopulations.

## Introduction

Lung cancer is one of the most frequent malignancies with 2.2 million new cases a year and the most common cause of cancer death in the world (1). Non-small cell lung cancer (NSCLC) is about 80–85% of all lung cancer cases (2). Majority (> 55%) of NSCLC patients are diagnosed late at the advanced stages of the disease (3). Currently, the treatment options for NSCLC are systemic therapies such as chemotherapies, drugs targeting commonly mutated pathways in lung cancer, and immune checkpoint inhibitors, surgical resection of the primary tumor or metastatic lesion and radiation therapy (4). The management and therapy of patients with advanced NSCLC have changed markedly in the last few years. Early detection methods and therapeutic options have been improved tremendously. However, the overall survival rate of patients with advanced NSCLC did not improve much and is still dismal (5). The reported 5-year survival rate of NSCLC patients was 17.8%, which is one of the highest fatality rates in non-communicable diseases (6). Reliable markers of treatment response and survival outcomes are urgently needed to enable early adaptation of treatment strategies in advanced NSCLC patients. Tumorigenesis and programmed cell death ligand 1 (PD-L1) status became standard markers influencing therapy regimes (7). Expression of PD-L1 has shown a significant predictive role (8). Other biomarkers such as lung immune prognostic index (LIPI) and tumor mutation burden (TMB) have revealed inconsistent results (9). Several studies have also demonstrated that lung cancer patients have lower levels of CD4+/CD8+ ratio, CD4+T cells, NK cell levels, and higher regulatory T cells (Tregs) than healthy subjects (10, 11). Patient-related factors such as age and comorbidities also affect peripheral blood lymphocyte subpopulations and treatment outcomes of NSCLC patients (12). Anti-cancer therapies, tumor surgery and wound healing may also have an impact on the immunophenotyping of lymphocyte subpopulations. So far, there are limited data concerning changes or developmental shifts in the most relevant B cell, T cell, and NK cell subpopulations caused by different anti-cancer therapies. Understanding the potential changes in lymphocyte subpopulations during different therapies is crucial to understand the effect of different therapies on the immune system and to adapt effective therapy regimens for better management of NSCLC patients. Therefore, in this study, we investigated the influence of immunotherapy, chemotherapy, immunochemotherapy, adjuvant chemotherapy after surgery, and antibodies against Vascular Endothelial Growth Factors (VEGF) on B cell, T cell, and NK cell subpopulations. Additionally, the relationship between therapy regimens and survival was assessed.

## Materials and methods

### Study design, population, and period

A longitudinal cohort study was performed on 32 consecutive NSCLC patients who visited the Pulmonology Clinic, Leipzig from January 10, 2018 to March 3, 2020. Socio-demographic information such as age, sex, smoking status, and clinical information such as type of cancer and the stage of cancer were collected from patients using a standardized questionnaire. Active smokers were defined as patients actively smoking at the time of data collection or up to 6 months before the data collection. To be qualified as former smoker, it was required that the patient quit smoking more than 6 months prior to data collection. Immunophenotyping was done before the administration of the planned therapy and during therapy with up to 7 observational windows (3 to 4 weeks each window) in the laboratory of the Institute of Clinical Immunology, University Hospital Leipzig resulting in a total of 135 observations with 130 immunologic parameters assessed. The median follow-up period for the study participants included in this study was 1,220 days.

### Ethical considerations

Ethical approval was made by the Medical Faculty of the University of Leipzig ethics committee (Ref. No: 201/18-ek). The socio-demographic information, clinical data, and venous blood were collected from NSCLC patients following the ethical considerations recommended by the Declaration of Helsinki of 1975, revised in 2013. Data identifying study participants were removed prior to analyses. Safeguards for appropriate and ethical use of data were in place. Informed consent was acquired from all NSCLC patients included in the study.

### Treatment options used

Patients were treated with one of the following five different treatment options: Immunotherapy (Nivolumab, Pembrolizumab, or Atezolizumab), Cytotoxic chemotherapy (Carboplatin + Nab-Paclitaxel, Carboplatin + Pemetrexed + Bevacizumab or Cisplatin + Pemetrexed + Bevacizumab), Immunochemotherapy (Carboplatin + Pemetrexed + Pembrolizumab, Carboplatin + Nab-Paclitaxel + Atezolizumab, Carboplatin + Nab-Paclitaxel + Pembrolizumab or Carboplatin + Nab-Paclitaxel + Bevacizumab + Atezolizumab), Adjuvant chemotherapy after tumor surgery (Cisplatin + Vinorelbin or Carboplatin + Vinorelbin) and Antibodies against VEGF (Bevacizumab).

### Blood collection

Ethylenediaminetetraacetic acid (EDTA) venous blood was collected from each patient in the pulmonology clinic before the administration of the planned therapy at the time of treatment start and during observational windows. EDTA blood specimen was immediately brought to the laboratory of the Institute of Clinical Immunology for analysis of the cellular immune status. Blood samples were processed and analyzed within 3 hours of collection.

### Cell surface markers staining

The staining and flow cytometry analysis of B cell, T cell, and NK cell subpopulations were performed according to previously published protocols (13, 14). In brief, whole blood sample was divided into 8 different panel tests to analyze the following specific cell populations: (i) general lymphocytes, (ii) B cell subsets, (iii) CD4 T cell subsets, (iv) CD8 T cell subsets, (v) regulatory T cells, (vi) recent thymic emigrants (RTEs), (vii) NK cell subsets, and (viii) NK cell activation markers. To each panel test, a 100 µl whole blood specimen was added and incubated with specific antibodies to the chosen cell subsets. The antibody mixes, cell markers, fluorochromes, IgG subtypes, antibody concentration, and clones used for every panel test are explained in **Supplementary Table S1**.

To decide the optimum dilution for each staining, each antibody for eight-color analysis panels was titrated separately and compared with isotype controls. Cell surface markers were stained for 15 minutes in the dark at room temperature and then erythrocytes were lysed by lysis buffer (BD Biosciences, Heidelberg, Germany) after incubation or 10 minutes. After centrifugation and washing with phosphate-buffered saline (PBS) (Biochrom, Berlin, Germany), cells were fixed with 200 µl PBS that contains 1% formaldehyde and ready for flow cytometry analysis.

### Flow cytometry analysis

The flowcytometry data acquisition was made using FACS Canto II (BD Biosciences) machine. The FACS Canto II is has three lasers: a 647 nm red laser, a 488 nm blue laser, and a 405 nm violet laser. During the analysis of all panel tests, automatic standard compensation was done, except for the B cell subsets panel, where the spectral overlap of the allophycocyanin (APC) toward peridinin-chlorophyll-protein (PerCP) was set to 12.66, to achieve optimal separation of memory and naive B cells. A total of 50,000 cell events were collected in the lymphocyte collection gate (CD45+ versus SSClow) for eight-color analysis, and the FACS DIVA (BD Biosciences) software version was used for data analysis 8. The absolute cell counts were determined using the relative flow cytometric cell counts measured by BD FACS Canto II Flow Cytometer set in relation to absolute cell counts analyzed by Sysmex XP-300 automated hematology analyzer (Sysmex Germany GmbH, Bornbarch, Germany) based on the standard protocol.

### General lymphocytes gating strategy

**Figure 1:**
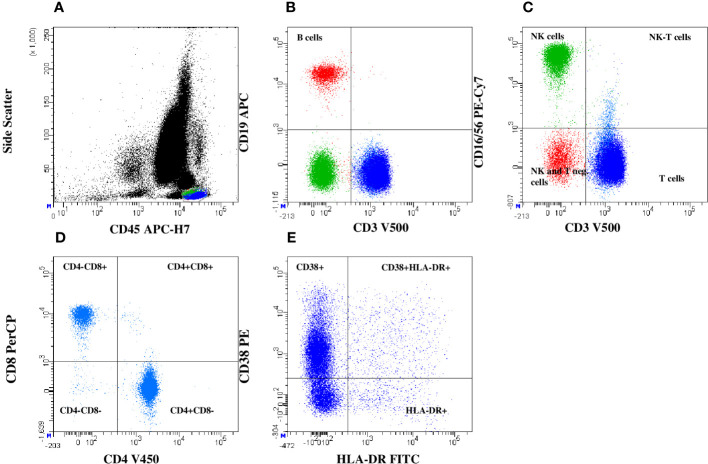
General lymphocytes gating strategy. Based on lymphocyte separation (SSC vs. CD45) **(A)**, T cells, B cells **(B)**, NK cells, and NK-T cells **(C)** were detected. From CD3+T cells gating, T helper (CD4+) cells, cytotoxic T cells (CD8+), CD4 and CD8 double positive and double negative T cells **(D)** and T cells activation status by HLA-DR+CD38+ expression were determined **(E)**.

The general lymphocyte gating strategy gives an overview of changes in the lymphocyte subpopulations. B cells (CD19+), T cells (CD3+), and NK cells (CD16+/56+) as well as HLA-DR+CD38+ activated T cells were gated from the lymphocyte gate using specific markers. Moreover, NK-T cells (T cells co-expressing CD16 molecule) were separated by gating CD3 versus CD16/56. The CD8 versus CD4 plot was used to separate 4 different T cell subsets: CD4+ and CD8+ T cells, CD4-/CD8- double-negative and CD4+/CD8+ double-positive T cells (**Figure 1**).

### B cell subsets gating strategy

B cells (CD19+) subsets were idetnified from the lymphocyte gate. The CD21 vs. CD27combination was used for the separation of immature, CD21low, and memory B cells. Double-negative immature B cells were used as a baseline for CD21+ or CD27+ B cells. Non-switched and switched memory B cells were separated from the CD27 gate by IgD versus IgM demarcation. The CD38low versus CD21low plot creates the gates for the activated CD21lowCD38low B cells and immature transitional B cells, which are specified to be CD21lowCD38+ B cells. IgM+ and CD38+ co-expressions were used for gating Transitional B cells. From this plot, plasma-blasts were separated using CD38+ expression and the absence of surface IgM. Additionally, CD27+CD138+ peripheral plasma cells were identified (**Supplementary Figure S1(I)**).

### T cell subpopulations gating strategy

T cells were separated from other lymphocytes using CD3 staining. To analyze T cell subpoulations, a combination of CD45RO, CD45RA, and CCR7 markers was used to separate effector cells (CD45RA+CD45RO-CCR7-), naive (CD45RA+CD45RO-CCR7+), central memory (CD45RA-CD45RO+CCR7+) and effector memory (CD45RA-CD45RO+CCR7-) cells. Additionally, from the CD3+CD4+ T helper cell population, regulatory T cells were detected as CD25+CD127 low cells (**Supplementary Figure S1(II)**).

### NK cells subsets gating strategy

For NK cells subsets analysis, T cells were excluded by gating the CD3 negative cells. From the CD3 negative cells, CD16 and CD56 markers were used to separate mature NK cells (CD16+CD56dim) and CD56 bright NK cells. In the next step, the expression of NK cells activation markers such as Nkp30, Nkp44, Nkp46, and CD57 was analyzed on mature NK cells (**Supplementary Table S1(III)**).

### Statistical analysis

Descriptive statistics were used to analyze demographic and clinical characteristics of patients before and after anti-cancer treatment. Treatment effects on the immunologic parameters compared to their baseline values were determined by applying linear mixed models (random intercept and random slope) with the statistical software suite R (version 4.2.0, R packages “lme4” and “lmerTest”) to consider multiple measurements per subject. Time points were assumed to be equidistant for this purpose. We further adjusted for age. Parameters were initially log- or logit-transformed if they were on an absolute or percentage scale, respectively. We corrected for multiple significance testing of changes in lymphocyte subpopulations after anti-cancer treatment by controlling the False Discovery Rate (FDR, Benjamini–Hochberg) for the 130 tests performed, so that FDR is less than or equal to 5%. In the following we denote the corrected p-values (according to FDR) as “q-values”. For survival analysis, the ratios of survivors to all included patients within each treatment option were calculated.

## Results

### Demographic and clinical characteristics of study participants

A total of 32 patients with NSCLC were enrolled in this study. Median age of patients included in this study was 68 years (range, 50–83 years), and 22 (68.7%) patients were ≥ 65 years old. The majority of study participants were male (N=24, 75%). The most frequent type of NSCLC, was squamous cell carcinoma in 16 (50%) of patients. Moreover, 20 (62.5%) patients had stage IV advanced NSCLC. Almost all patients 30 (96.9%) were current or former smokers. Immunochemotherapy was the most commonly administered therapy (N=12, 37.5%) followed by cytotoxic chemotherapy (N=8, 25%), adjuvant chemotherapy after surgery (N=7, 21.9%), and immunotherapy (N=4, 12.5%) (**Table 1**).

**Table 1:** Socio-demographic and clinical characteristics of study participants by treatment group (N = 32).

Characteristics		Treatment groups						Total (N=32) N (%)
		Immuno-therapy (N=7)	Chemo-therapy (N=7)	Immuno-chemo- therapy (N=9)	Adjuvant chemo- therapy (N=5)	Antibodies against VEGF (N=1)	Baseline (N=3)	
Age group (years)	< 65	3	2	2	2	1	0	10 (31)
	> 65	4	5	7	3	0	3	22 (69)
	Median	67	69	68	65	59	79	68.5
Sex	Male	6	4	6	4	1	3	24 (75)
	Female	1	3	3	1	0	0	8 (25)
Smoking status	Active smoker	3	3	4	4	0	1	15 (47)
	Former smoker	4	4	4	1	1	2	16 (50)
	Non-smoker	0	0	1	0	0	0	1 (3)
NSCLC type	Squameus cell carcinoma	5	3	2	4	0	2	16 (50)
	Adenocarcinoma	1	4	7	1	1	1	15 (47)
	Non specific carcinoma	1	0	0	0	0	0	1 (3)
Cancer stage	IA3	0	0	0	1	0	0	1 (3)
	IIB	0	1	0	2	0	1	4 (13)
	IIIA	0	0	2	1	0	0	3 (9)
	IIIB	0	0	0	1	0	1	2 (6)
	IIIC	1	1	0	0	0	0	2 (6)
	IVA	4	2	2	0	0	0	8 (25)
	IVB	2	3	5	0	1	1	12 (38)

NSCLC, Non-small cell lung cancer; VEGF, Vascular endothelial growth factors; N, number. Baseline = Measurement before therapy initiation.

### Effects of anti-cancer therapies on B cell subpopulations

**Figure 2:**
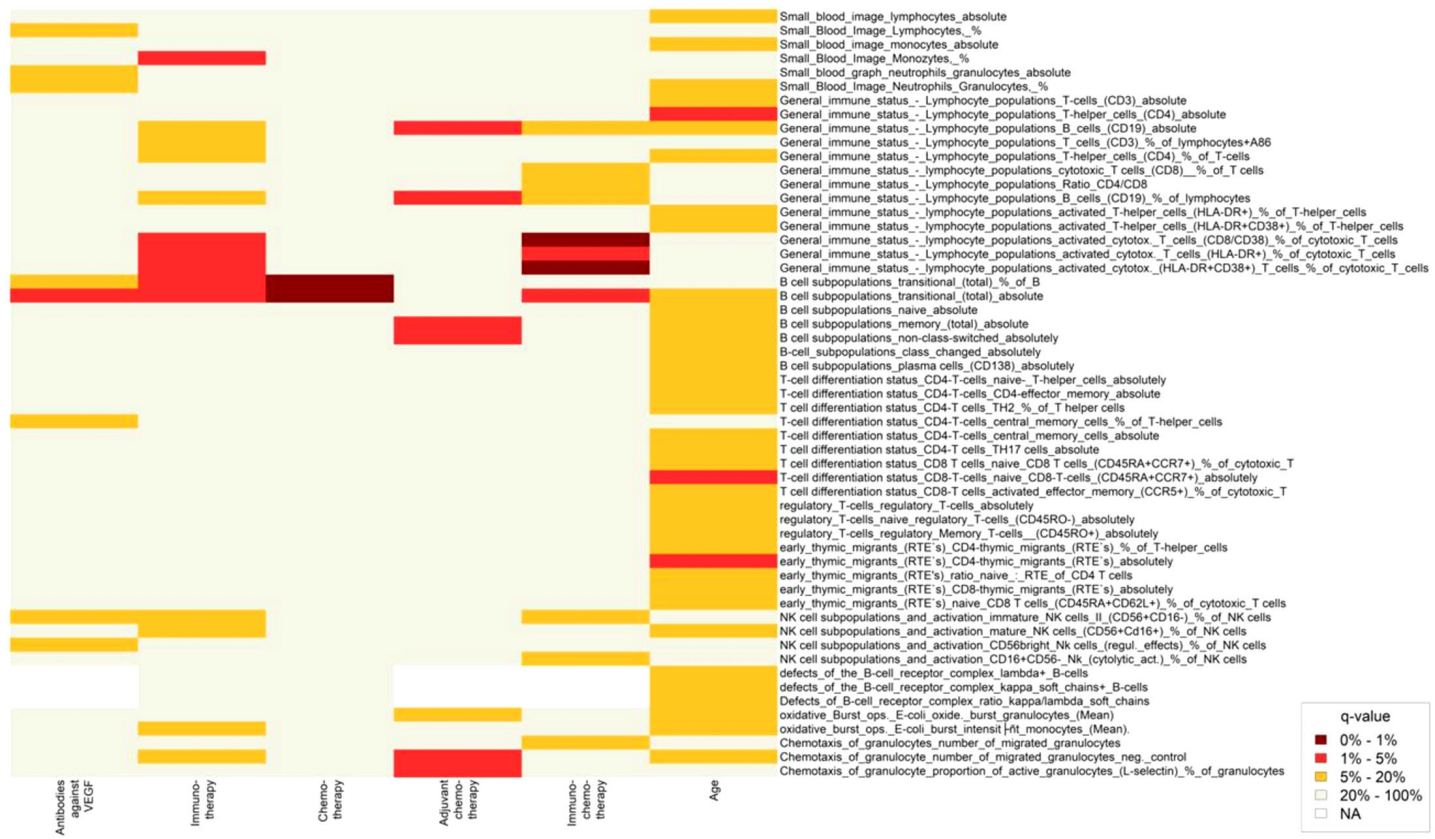
Heatmap showing changes in lymphocyte subpopulations after treatment with different anti-cancer therapies and aging. Statistically significant changes in lymphocyte subpopulations are indicated by dark red and red color boxes (FDR = 0 - 1% and 1 - 5%, respectively).

Absolute number of transitional B cells was significantly increased after therapy with immunotherapy (q=0.032), immunochemotherapy (q=0.030), and antibodies against VEGF (q=0.024). The transitional B cells percentage was significantly increased after immunotherapy (q=0.033). The percentage of B cells and absolute number of B cells were significantly increased after adjuvant chemotherapy (q=0.023). However, the percentage and absolute number of transition cells were significantly decreased after chemotherapy (q=0.001). Likewise, the absolute number of memory B cells and non-switched B cells were decreased significantly after adjuvant chemotherapy (q=0.023, and q=0.018, respectively) (**Table 2**, **Figure 2**).

**Table 2:** Changes in B cell subpopulations in NSCLC patients during different anti-cancer therapies.

B cell subpopulations	Immunotherapy			Chemotherapy			Immuno- chemotherapy			Adjuvant chemotherapy			Antibodies against VEGF		
	before	after	q-value	before	after	q-value	before	after	q- value	before	after	q-value	before	after	q-value
B cells (cells/µL)	111.4	75.3	0.064	101.6	165.1	0.775	132.7	73.0	0.088	273.0	373.6	0.023	99	162	0.732
B cells % of lymphocytes	9.1	7.0	0.057	11.7	9.7	0.839	10.3	6.6	0.159	14.6	20.2	0.023	9	9	0.856
Immature % of B cells	9.8	8.1	0.896	5.7	7.0	0.892	8.6	10.7	0.815	7.0	7.3	0.566	4.1	2.6	0.856
Immature B cells (cells/µL)	10.1	5.9	0.525	5.9	11.3	0.754	12.3	6.5	0.336	6.9	16.3	0.524	4.1	4.2	0.856
Transitional B cells % of B cells	1.1	7.8	0.033	2.2	0.4	0.001	1.2	1.1	0.209	0.7	0.0	0.450	1.3	9.9	0.175
Transitional B cells (cells/µL)	1.1	7.4	0.032	1.9	1.1	0.001	1.3	1.5	0.030	1.2	0.0	0.681	1.3	16	0.024
Immature transitional B cells % of B cells	6.7	1.4	0.848	15.5	2.2	0.775	NA	NA	NA	58.0	82.5	0.524	NA	NA	NA
Immature transitional B cells (cells/µL)	2.5	0.2	0.800	11.3	4.5	0.897	NA	NA	NA	30.2	280.5	0.450	NA	NA	NA
Naive % of B cells	64.6	65.1	0.951	67.9	69.8	0.775	55.9	56.8	0.674	39.0	28.4	0.566	86.2	90	0.732
Naive B cells (cells/µL)	66.8	52.4	0.885	70.5	103.9	0.986	60.4	41.2	0.548	100.4	58.0	0.422	85.3	145.8	0.732
Memory % of B cells	15.3	17.3	0.896	7.8	14.0	0.893	22.3	18.7	0.629	32.3	30.0	0.493	4.8	3.3	0.732
Memory B cells (cells/µL)	23.3	10.7	0.743	9.5	33.8	0.839	37.5	14.9	0.318	148.7	57.4	0.023	4.8	5.3	0.988
Non-class-switched % of B cells	3.7	3.8	0.896	1.3	2.2	0.897	6.6	4.7	0.974	24.4	16.5	0.334	2	1.2	0.732
Non-class-switched B cells (cells/µL)	7.3	2.5	0.987	1.4	5.4	0.775	12.8	3.7	0.606	135.9	35.6	0.018	2	1.9	0.936
Class-switched % of B cells	8.9	11.1	0.903	6.7	10.3	0.893	172.2	10.4	0.499	6.0	10.3	0.875	2.5	1.9	0.856
Class-switched B cells (cells/µL)	13.5	6.7	0.896	8.5	24.5	0.839	11.5	8.2	0.345	6.4	15.1	0.334	2.5	3.1	0.907
CD21low % of B cells	3.2	5.5	0.477	2.5	3.4	0.865	NA	NA	NA	NA	NA	NA	NA	NA	NA
CD21low B cells (cells/µL)	1.3	0.9	0.743	3.2	8.4	0.897	NA	NA	NA	NA	NA	NA	NA	NA	NA
Activated CD21low CD38low % of B cells	2.2	3.5	0.650	1.7	2.1	0.893	NA	NA	NA	NA	NA	NA	NA	NA	NA
Activated CD21low CD38low B cells (cells/µL)	0.9	0.6	0.885	2.4	5.1	0.775	NA	NA	NA	NA	NA	NA	NA	NA	NA
Plasmablasts (CD38) % of B cells	1.4	1.2	0.987	0.8	4.5	0.588	2.0	2.2	0.590	0.9	1.4	0.831	0.2	0.2	0.856
Plasma cells (CD38) (cells/µL)	2.7	1.0	0.553	0.7	8.3	0.754	3.3	1.8	0.559	0.9	2.4	0.546	0.2	0.3	0.856
Plasma cells (CD138) % of B cells	1.5	1.0	0.903	0.7	2.1	0.955	1.7	2.2	0.227	1.5	1.2	0.833	1.5	0.4	0.880
Plasma cells (CD138) (cells/µL)	1.0	0.7	0.743	0.6	3.5	0.839	2.9	1.7	0.987	1.8	2.3	0.488	1.5	0.6	0.732

Values “before” and “after” correspond to the measured values before the initial treatment and the measured value after the last treatment, respectively, after an average observation time of 718/459/473/1,060/1,462 days of immunotherapy/chemotherapy/immunochemotherapy/adjuvant chemotherapy/antibodies against VEGF. Q-values correspond to the significance of the estimated change over time from linear mixed modelling. VEGF, Vascular endothelial growth factors; NA, Not available.

### Effects of anti-cancer therapies on T cell subpopulations

After immunotherapy, a significant increase was observed in activated cytotoxic T cells (CD8/CD38) (q=0.050), activated cytotoxic T cells (HLA-DR+) (q=0.031), and activated cytotoxic (HLA-DR+CD38+) T cells (q=0.031). Similarly, after immunochemotherapy, a significant increase was revealed in activated cytotoxic T cells (CD8/CD38) (q<0.0001), activated cytotoxic T cells (HLA-DR+) (q=0.030), and activated cytotoxic (HLA-DR+CD38+) T cells (q=0.002). There was a trend that the percentage of cytotoxic T cells (CD8+) and T cells (CD3+) were increased after immunotherapy (q=0.057) and immunochemotherapy (q=0.080), respectively. Chemotherapy, adjuvant chemotherapy, and antibodies against VEGF therapies did not induce significant changes in numbers of T cell subpopulation and activation (**Table 3**, **Supplementary Table S2**, **Figure 2**).

**Table 3:** Changes in T cell subpopulations in NSCLC patients after different anti-cancer therapies.

T cell subpopulations	Immunotherapy			Chemotherapy			Immuno-chemotherapy			Adjuvant chemotherapy			Antibodies against VEGF		
	before	after	q-value	before	after	q-value	before	after	q-value	before	after	q-value	before	after	q-value
T cells (cells/µL)	575.7	952.6	0.896	631.7	1099.1	0.839	946.1	811.1	0.548	983.2	1131.4	0.673	869	1530	0.841
T helper cells (cells/µL)	336.3	546.0	0.985	429.0	758.7	0.981	652.7	577.8	0.503	628.6	706.0	0.646	583	954	0.749
Cytotoxic T cells (cells/µL)	173.3	334.9	0.743	158.1	266.7	0.882	219.7	194.4	0.618	268.8	352.4	0.566	242	504	0.789
T cells % of lymphocytes+A86	63.9	72.9	0.057	67.9	69.4	0.588	73.4	72.8	0.994	71.0	66.8	0.605	79	85	0.934
T helper cells % of T cells	37.1	43.7	0.111	45.3	40.3	0.814	51.1	51.4	0.590	45.0	41.8	0.708	53	53	0.907
Cytotoxic T cells % of T cells	21.7	24.0	0.848	17.9	23.3	0.588	16.9	17.8	0.080	19.8	20.6	0.781	22	28	0.732
CD4/CD8 ratio	2.4	2.6	0.487	3.6	2.5	0.897	3.8	3.3	0.159	2.3	2.1	0.877	2.4	1.9	0.749
Double negative T cells (CD4-CD8-) % of T cells	6.2	4.9	0.920	2.1	4.0	0.986	5.3	4.2	0.318	5.5	4.6	0.697	4.7	3.6	0.907
Double positive T cells (CD4+CD8+) % of T cells	0.7	1.6	0.729	4.0	3.7	0.839	1.1	1.2	0.953	2.2	1.7	0.781	0.9	1.2	0.856
Activated T-helper cells (CD38+) % of T-helper cells	53.7	50.4	0.987	41.6	44.9	0.839	66.1	68.4	0.326	44.0	47.0	0.784	46	46	0.732
Activated T-helper cells (HLA-DR+) % of T-helper cells	15.9	15.4	0.431	10.7	16.1	0.893	8.6	9.9	0.807	15.8	10.6	0.566	11	7	0.856
Activated T helper cells (HLA-DR+CD38+) % of T helper cells	9.3	7.4	0.525	4.1	9.9	0.588	4.0	6.5	0.330	6.6	5.8	0.903	4	3	0.907
Activated cytotoxic T cells (CD8/CD38) % of cytotoxic T cells	44.4	57.4	0.050	32.4	38.0	0.588	34.9	51.3	0.000	31.8	40.2	0.546	36	49	0.856
Activated cytotoxic T cells (HLA-DR+) % of cytotoxic T cells	26.9	38.1	0.031	19.1	35.4	0.588	14.7	19.5	0.030	37.6	42.2	0.566	40	52	0.732
Activated cytotoxic (HLA-DR+CD38+) T cells % of cytotoxic T cells	17.7	29.7	0.031	10.1	23.7	0.588	7.6	14.4	0.002	18.8	26.8	0.334	13	32	0.732

Values “before” and “after” correspond to the measured values before the initial treatment and the measured value after the last treatment, respectively, after an average observation time of 718/459/473/1,060/1,462 days of immunotherapy/chemotherapy/immunochemotherapy/adjuvant chemotherapy/antibodies against VEGF. Q-values correspond to the significance of the estimated change over time from linear mixed modelling. VEGF, Vascular endothelial growth factors; HLA-DR, Human Leukocyte Antigen-DR isotype.

### Effects of anti-cancer therapies on NK cells subsets

In our study, none of the treatment options used had a statistically significant effect on NK cell subsets. However, an increased trend in the percentage of immature NK cells II (CD56+CD16-) after immunotherapy (q=0.057) and a decreased trend in the percentage of CD16+CD56 NK cells (cytolytic activity) after immunochemotherapy (q=0.059) was observed (**Table 4**; **Figure 2**).

**Table 4:** Changes in the NK cell subpopulations in NSCLC patients after different anti-cancer therapies.

NK cell subpopulations	Immunotherapy			Chemotherapy			Immuno- chemotherapy			Adjuvant chemotherapy			Antibodies against VEGF		
	before	after	q-value	before	after	q-value	before	after	q-value	before	after	q-value	before	after	q-value
NK cells (CD16/56) (cells/µL)	238.1	180.7	0.219	174.4	205.9	0.588	198.6	193.6	0.659	199.0	210.0	0.697	123	104	0.841
NK cells (CD16/56) % of lymphocytes	26.5	19.6	0.232	19.6	20.4	0.588	15.7	19.5	0.583	14.2	12.7	0.334	11.2	5.8	0.732
NK T cells (CD3/56) % of lymphocytes	6.4	5.2	0.801	6.3	5.1	0.865	6.1	5.6	0.499	5.7	3.7	0.508	4.3	3.1	0.856
Immature NK cells I (CD56-CD16-) % of NK cells	0.1	0.2	0.243	0.3	0.5	0.882	0.4	0.4	0.953	0.4	0.2	0.857	1.7	0.9	0.732
Immature NK cells II (CD56+CD16-) % of NK cells	3.3	4.4	0.057	3.6	4.2	0.588	4.5	5.4	0.159	3.2	4.3	0.508	14.7	13.9	0.122
Mature NK cells (CD56+Cd16+) % of NK cells	90.8	81.8	0.158	83.5	85.5	0.991	84.6	84.5	0.949	77.9	76.3	0.392	69.8	57.2	0.732
CD56bright Nk cells (regulatory effects) % of NK cells	2.8	4.4	0.729	2.9	3.7	0.588	2.9	3.5	0.548	3.1	6.0	0.546	1.3	7.6	0.130
CD16+CD56- Nk cells (cytolytic activity) % of NK cells	4.2	11.2	0.920	10.5	7.7	0.775	8.3	7.6	0.059	15.9	15.3	0.718	7.1	21.3	0.856
Activated CD94/NKG2D complex % of NK cells	36.0	39.4	0.985	31.7	27.9	0.853	42.0	41.2	0.629	32.4	41.4	0.833	8.9	28.8	0.856
Nkp30% of NK cells	81.1	73.7	0.800	76.8	76.3	0.897	61.6	79.4	0.930	62.8	57.1	0.566	77.8	63.1	0.732
Nkp46% of NK cells	62.7	57.9	0.985	46.5	53.2	0.865	57.1	64.0	0.618	44.5	42.3	0.711	44.6	46.2	0.856
Nkp44 (cytolytic activity) % of NK cells	0.5	0.6	0.920	5.1	1.2	0.588	0.6	0.8	0.953	2.1	0.6	0.780	0.1	0.7	0.907
CD57% of NK cells	47.7	43.2	0.650	44.1	44.0	0.977	55.0	55.6	0.604	42.9	39.4	0.646	56.6	43.3	0.732

VEGF, Vascular endothelial growth factors.

### Effects of anti-cancer therapies on monocytes and granulocytes

In addition to lymphocytes, we assessed possible changes in monocytes and granulocytes after our therapy options. Percentages of monocytes significantly increased from 3.7% to 11.7% (q=0.033) after immunotherapy. Moreover, in the chemotaxis of granulocytes, the number of migrated granulocytes was significantly increased (q=0.023), while chemotaxis of granulocytes and percentages of active granulocytes (L-selectin) significantly decreased (q=0.018) after adjuvant chemotherapy. Other therapy options did not show significant changes in monocyte and granulocyte parameters (**Supplementary Table S2**).

### Effect of age on T cell, B cell, and NK cell subpopulations

Age showed significant association with CD4+ T-lymphocytes, naive (CD45RA+CCR7+) CD8+T cells, and CD4- recent thymus migrations cells (q<0.05). However, age was not significantly associated with lymphocyte subpopulations showing significant changes after anti-cancer therapies. These findings suggest that the observed changes in lymphocyte subpopulations after anti-cancer therapies are not confounded by age (**Figure 2**, **Supplementary Figure S2**).

### Survival of NSCLC patients

Overall, 9/29 (31%) of the patients survived. For adjuvant chemotherapy we observed that 4/5 (80%) of patients survived. Patients with immunotherapy and immunochemotherapy achieved comparable survival with 2/7 (29%) and 2/9 (22%), respectively. Within chemotherapy 0/7 (0%) of patients survived in contrast to the patient who received therapy with antibodies against VEGF (1/1 (100%)).

## Discussion

This study was aimed to analyse the impact of different therapeutic regimens on the immune system of NSCLC patients investigating changes in B-, T-, and NK cell subsets during therapy.

The absolute numbers of transitional B cells were significantly increased after therapy with immunotherapy, immunochemotherapy, and antibodies against VEGF. Percentages of transitional B cells also significantly increased after immunotherapy. Similarly, absolute numbers and percentages of B cells were significantly increased after adjuvant chemotherapy. The observed upregulation of B cells and transitional B cells during these therapies suggest activation of immune defense. These effects of therapies on B cells also imply the potential use of these cells as a target for vaccination against lung cancer. In line with this, targeted strategies to enhance B cells by using stimulated B cell ligands have been applied to inhibit lung metastasis and tumor growth (15, 16).

B cells are important immune cells that support an effective antitumor immune response by their capability to present tumor antigens, activate cytotoxic T cell and T-lymphocytes, response, as well as their ability to generate cytokines and anti-tumor antibodies (17–19). In line with the findings of this study, previous studies suggest that B cell counts increase in tumors of patients responding to immunotherapy, and tumor-infiltrating B cells were associated with, tumor stage and longer survival in NSCLC (20, 21). Even though the importance of B cells in making anti-cancer immune responses was progressively unraveled in the last years, a better understanding about the role of different B cell subsets, particularly transitional B cells is still required in cancer patients (17).

Interestingly, absolute numbers and percentages of transitional B cells significantly decreased after chemotherapy. Similarly, the absolute number of memory B cells and non-class switched B cells significantly decreased after adjuvant chemotherapy. Down-regulation of B cells during chemotherapy suggests a potential suppression of immune defense due to cytotoxic side-effects on the bone marrow. A previous study also reported that chemotherapy reduced the absolute counts of B cells (22). Another study showed that chemotherapy reduced absolute counts of B cells followed by a slow increase but not reaching baseline levels. This suggests that the chemotherapy has a long-term effect on lymphocyte subsets (23).

In this study, we noticed a significant increase in activated cytotoxic T cells in patients treated with immunotherapy and immunochemotherapy. The immune system activation by immunotherapy and immunochemotherapy is a desired therapeutic effect. T lymphocytes are the most important inflammatory cells that infiltrate the tumor, exert a direct cytotoxic effect, or cause lysis of tumor by cytokine release (24–26). CD8+ T cells can also increase and differentiate into CD8+ T cells that can infiltrate tumors by peripheral blood migration and play an essential role in the direct killing of tumor cells (27). Our finding is in line with the reported increase of CD8+ T cells after immunotherapy in lung cancer patients (28). Another study also suggested that as part of a successful immunotherapy response, effector memory tumor antigen-specific cytotoxic T cells are increased (29). However, in contrast to our findings, other studies reported decreased proportion of CD8+ T cells after immunotherapy as compared to baseline values (30, 31).

The observed differences in cytotoxic T cell response after immunotherapy could be explained as follows. First, lung cancer patients treated with PD-1 inhibitors are characterized by expressing effector-like phenotypes (HLA-DR+, CD38+, Bcl-2lo), co-stimulatory molecules (CD28, CD27, ICOS), a highly specific subset of proliferating CD8+ T cells in the peripheral blood, and high levels of PD-1 (32). Second, different timings of blood sampling could contribute to the observed contradictory findings across studies. CD8+ T cell response induction by blocking the PD-1 pathway in the peripheral blood is transient and detected during the first four weeks after treatment start. Then these specific CD8+ T cells migrate to the tumor sites (32).

Although none of the therapies investigated in this study showed a significant effect on NK cells, a trend increase in the percentage of immature NK cells was observed after immunotherapy. A decrease in the percentage of CD16+CD56 NK cells was also observed after immunochemotherapy. A previous study demonstrated that the PD-L1 leads blockage of causes activation of NK cells and increases the direct antitumor effect of NK cells (33). Another study showed that in NSCLC patients, NK cell activity is a marker to predict the immunotherapy response (34). Drugs modulating the function and proliferative activity of NK cells have been developed to produce synergistic effect in combination with immunotherapy (35, 36).

In addition to lymphocyte subpopulations, we assessed the possible effects of anti-cancer therapies on monocytes and granulocytes. Percentage of monocytes showed a significant increase after immunotherapy. This could be interpreted as the effect of a T cell interaction and activation by immunotherapy as described in a previous study (37). In several tumor types, an increase in HLA-DR low monocytes has been reported (38). As already described for HLA-DR low monocytes in sepsis, these monocytic cells might decrease T cell function in patients with cancer (39). Moreover, the number of migrated granulocytes in this chemotaxis study was significantly increased, while the percentage of active granulocytes (L-selectin) decreased after adjuvant chemotherapy. This is possibly due to the consequence of high baseline activity, postoperative inflammation, and wound healing processes. Previous studies also reported that the number of neutrophils in tumor tissues and blood is correlated with poor patient outcomes and disease progression (40). Neutrophil count was associated with the percentage of HLA-DR low monocytes in patients with lung cancer managed by surgery of the primary tumor as an important subpopulation of the myeloid-derived suppressor cells (MDSC). Nevertheless, in the late tumor stages, this observation was not confirmed (41).

In the present study, NSCLC patients treated with adjuvant chemotherapy showed the highest survival of 80%. In contrast, patients treated with chemotherapy alone showed the lowest survival with 0%. This could be explained by the fact that adjuvant chemotherapy is usually applied for early-stage NSCLC; stage IIA, IIB, and IIIA NSCLC to kill remaining cancer cells after surgery (42). Adding immunotherapy to chemotherapy in a palliative setting increased observed survival to 22% compared to chemotherapy alone.

This is consistent with previous reports showing a significant increase in overall response rates, overall survival and progression-free survival in NSCLC patients treated with immunotherapy compared to chemotherapy (43). Five-year follow-up study showed a significantly increased median time of overall survival, which doubled from 13.4 months in chemotherapy to 26.3 months in immunotherapy; and a higher overall survival rate, 31.9% in the immunotherapy group compared to 16.3% in the chemotherapy group (44). Immunotherapy enhances the immune system that targets cancer cells and slows or stops the cancer cells metastasis and growth (45). Previous studies also showed that improved survival is correlated with a strong immune response to tumers. Moreover, higher numbers of CD8+ T cells and natural killer cells are linked with better patient survival (46, 47).

We repeated all the analyses twice, with further adjustment for tumor stage, and with further adjustment for tumor stage and exclusion of patients with adjuvant therapy to check that stage of tumor did not bias our initial analysis with adjustment for age only. Indeed, the results were comparable yielding no significant additional information.

This study has some limitations to be mentioned. Firstly, despite multiple measurements, due to the limited sample size our study might be underpowered to identify smaller changes in the lymphocyte subpopulations before and after treatment with different anti-cancer therapies. Likewise, survival analysis is limited by the small sample size. Secondly, this study was conducted at a single health care facility, therefore, selection bias cannot be fully excluded.

Since this study was the first one investigating such a large spectrum of parameters in cytomics, further research is needed to determine prognostic significance, further potential confounding factors and long-term survival.

## Data availability statement

The original contributions presented in the study are included in the article/**Supplementary Material**, further inquiries can be directed to the corresponding author/s.

## Ethics statement

The studies involving humans were approved by ethics committee of the University of Leipzig. The studies were conducted in accordance with the local legislation and institutional requirements. The participants provided their written informed consent to participate in this study.

## Author contributions

Conceptualization, PG, CG, MS, US. Methodology, PG, CG, US. Formal analysis, AK, BT. Investigation, PG, AB. Data curation, PG, CG, AK, BT. Original draft preparation, BT. Review and editing, PG, CG, US, AK, BT, AB, MS. Project administration, PG, CG. All authors contributed to the article and approved the submitted version.
