# Supplementary material for: The influence of anti-cancer therapies on lymphocyte subpopulations of lung cancer patients
Source: Front Immunol. 2023 Aug 28;14:1239097. doi: 10.3389/fimmu.2023.1239097 (PMC10493868; doi:10.3389/fimmu.2023.1239097)
Supplement: Supplementary file 1 [file DataSheet_1.pdf]

Supplementary Material

The influence of anti-cancer therapies on lymphocyte subpopulations of lung cancer patients

Philipp Gessner[1,2]†, Belay Tessema[1,3]†, Markus Scholz[4], Ulrich Sack[1], Andreas Boldt[1], Andreas Kühnapfel[4#], Christian Gessner*[1,5#]

† These authors share first authorship

# These authors share senior authorship

[1]Institute of Clinical Immunology, Faculty of Medicine, University of Leipzig, Leipzig, Germany

[2]Department of Respiratory Medicine, University Hospital Leipzig, Leipzig, Germany

[3]Department of Medical Microbiology, College of Medicine and Health Sciences, University of Gondar, Ethiopia

[4] Institute for Medical Informatics, Statistics and Epidemiology (IMISE), University of Leipzig, Leipzig, Germany.

[5] Pulmonary Practice, Leipzig, Germany

*** Correspondence:**
Christian Gessner
ch.gessner@pneumologe-leipzig.de

Supplementary Figures and Tables

Supplementary Table S1: The list of cell markers, fluorochromes, and IgG subtypes used for the identification of lymphocyte subpopulations.

**Panel**	**Molecular marker**	**Fluorochrome**	**Clone**	**IgG subtype**	**Antibody/100 ml blood**	**Company**
**Panel (i): general lymphocytes**						
	CD45	APC-H7	2D1	mouse IgG1	2.5 ml	BD Pharmingen
	CD3	V500	UCHT1	mouse IgG1	2.5 ml	BD Horizon
	CD4	V450	RPA-T4	mouse IgG1	2.5 ml	BD Horizon

	CD8	PerCP	SK1	mouse IgG1	5 ml	BD Biosciences
	CD19	APC	SJ25C1	mouse IgG1	2.5 ml	BD Biosciences
	CD16/56	PE-Cy7	CD16: B73.1<br><br>CD56: NCAM 16.2	CD16:Mouse IgG1<br><br>CD56:Mouse IgG2b	2.5 ml	BD Biosciences
	CD38	PE	HB7	mouse IgG1	5 ml	BD Biosciences
	Anti-HLA-DR	FITC	L243	mouse IgG2a	5 ml	BD Biosciences
**Panel (ii): B cell subsets**						
	IgD	FITC	IA6-2	mouse IgG2a	5 ml	BD Pharmingen
	CD21	PE	B-ly4	mouse IgG1	5 ml	BD Pharmingen
	CD27	PerCP Cy5.5	M-T271	mouse IgG1	2.5 ml	BD Pharmingen
	CD38	PE-Cy7	HB7	mouse IgG1	2.5 ml	BD Biosciences
	IgM	APC	G20–127	mouse IgG1	10 ml	BD Pharmingen
	CD45	APC-H7	2D1	mouse IgG1	2.5 ml	BD Pharmingen
	CD138	V450	MI15	mouse IgG1	2.5 ml	BD Horizon
	CD19	V500	HIB19	mouse IgG1	2.5 ml	BD Horizon
**Panel (iii): CD4 cell subsets**						
	CD45RA	FITC	HI100	mouse IgG2b	5 ml	BD Pharmingen
	CCR3	PE	5E8	mouse C57BL/6 IgG2b	2.5 ml	BD Pharmingen

	CD45RO	PerCP Cy5.5	UCHL1	mouse BALB/c IgG2a	2.5 ml	BD Pharmingen
	CCR5	PE-Cy7	2D7/CCR5	mouse C57BL/6 IgG2a	2.5 ml	BD Pharmingen
	CCR7	Alexa 647	3D12	Rat IgG2a	2.5 ml	BD Pharmingen
	CD45	APC-H7	2D1	mouse IgG1	2.5 ml	BD Pharmingen
	CD4	V450	RPA-T4	mouse IgG1	2.5 ml	BD Horizon
	CD3	V500	UCHT1	mouse IgG1	2.5 ml	BD Horizon
**Panel (iv): CD8 cell subsets**						
	CD45RA	FITC	HI100	mouse IgG1	5 ml	BD Pharmingen
	CCR5	PE	2D7/CCR5	mouse C57BL/6 IgG2a	5 ml	BD Pharmingen
	CD45RO	PerCP Cy5.5	UCHL1	mouse IgG1	2.5 ml	BD Pharmingen
	CCR6	PE-Cy7	11A9	mouse IgG1	2.5 ml	BD Pharmingen
	CCR7	Alexa 647	3D12	mouse IgG1	2.5 ml	BD Pharmingen
	CD45	APC-H7	2D1	mouse IgG1	2.5 ml	BD Pharmingen
	CD8	V450	RPA-T8	mouse IgG1	2.5 ml	BD Horizon
	CD3	V500	UCHT1	mouse IgG1	2.5 ml	BD Horizon
**Panel (v): regulatory T cells**						
	HLA-DR	FITC	L243	mouse IgG2a	5 ml	BD Biosciences
	CD25	PE	2A3	mouse BALB/c IgG1	5 ml	BD Biosciences
	CD45RO	PerCP Cy5.5	UCHL1	mouse IgG1	2.5 ml	BD Pharmingen

	CD127	Alexa 647	HIL-7R-M21	mouse IgG1	5 ml	BD Pharmingen
	CD45	APC-H7	2D1	mouse IgG1	2.5 ml	BD Pharmingen
	CD4	V450	RPA-T4	mouse IgG1	2.5 ml	BD Horizon
	CD3	V500	UCHT1	mouse IgG1	2.5ml	BD Horizon
**Panel (vi): recent thymic emigrants (RTE)**						
	TCR α/β	FITC	WT31	-	5 ml	BD Biosciences
	CD31	PE	WM59	mouse IgG1	5 ml	BD Pharmingen
	CD45RA	PerCP Cy5.5	HI100	mouse IgG2b	5 ml	eBioscience
	CD62L	PE-Cy7	DREG-56	mouse IgG1	10 ml	Biolegend
	TCR g/d	APC	B1	mouse IgG1	5 ml	Biolegend
	CD45	APC-H7	2D1	mouse IgG1	2.5 ml	BD Pharmingen
	CD4	V450	RPA-T4	mouse IgG1	2.5 ml	BD Horizon
	CD3	V500	UCHT1	mouse IgG1	2.5 ml	BD Horizon
**Panel (vii): NK cell subsets**						
	CD94	FITC	HP-3D9	mouse B12B/c IgG1	5 ml	BD Pharmingen
	CD314	PE	1D11	mouse IgG1	5 ml	eBiosciene
	CD161	PerCP Cy5.5	HP-3G10	mouse IgG1	2.5 ml	eBiosciene
	CD56	PE-Cy7	NCAM16.2	mouse IgG1	2.5 ml	BD Biosciences
	CD122	APC	TU27	mouse IgG1	5 ml	Biolegend
	CD45	APC-H7	2D1	mouse IgG1	2.5 ml	BD Pharmingen

	CD16	V450	3G8	Mouse BALB/C x DBA/2 IgG1	2.5 ml	BD Horizon
	CD3	V500	UCHT1	mouse IgG1	2.5 ml	BD Horizon
**Panel (viii): NK cell activation**						
	CD57	FITC	HNK1	mouse IgM	5 ml	BD Biosciences
	Nkp30	PE	P30-15		5 ml	BD Pharmingen
	CD16/56	PE-Cy7	CD16: B73.1<br><br>CD56: NCAM 16.2	mouse IgG1	2.5 ml	BD Biosciences
	Nkp44	Alexa 647	p44-8	Mouse BALB/c IgG1	10 ml	BD Pharmingen
	CD45	APC-H7	2D1	mouse IgG1	2.5 ml	BD Pharmingen
	Nkp46	V450	9E2/Nkp46	mouse IgG1	2.5 ml	BD Horizon
	CD3	V500	UCHT1	mouse IgG1	2.5 ml	BD Horizon

Supplementary Table S2: Changes in the proportion of leukocytes before and after treatment by different treatment options in NSCLC patients.

**Leukocytes**	**Antibodies against VEGF**			**Immunotherapy**			**Chemotherapy**			**Adjuvant chemotherapy**			**Immuno-chemotherapy**		
	before	after	q-value	before	after	q-value	before	after	q-value	before	after	q-value	before	after	q-value
**Blood Leukocytes count**															
Leukocytes (×10^9/L)	5.5	3.3	0.732	9.0	8.7	0.903	9.8	8.7	0.588	7.5	10.0	0.524	10.0	8.3	0.499
Lymphocytes (cells/µL)	1.1	1.8	0.789	0.9	1.2	0.650	0.9	1.5	0.775	1.5	1.7	0.508	1.3	1.1	0.499
Lymphocytes (%)	20.4	55.8	0.175	10.5	16.4	0.885	11.2	16.9	0.897	19.5	18.7	0.857	13.3	15.5	0.953
Monocytes (cells/µL)	0.4	0.4	0.933	0.4	0.9	0.441	0.9	0.7	0.835	1.1	1.0	0.831	0.8	0.9	0.548
Monocytes (%)	7.7	13.1	0.732	3.7	11.6	0.033	10.2	9.2	0.955	14.6	9.9	0.877	7.7	11.9	0.629
Neutrophil granulocytes (cells/µL)	4	1.1	0.134	7.2	5.7	0.743	8.1	6.5	0.588	4.9	7.3	0.566	7.9	6.1	0.590
Neutrophil granulocytes (%)	71.9	31.1	0.130	85.3	69.7	0.501	77.2	73.3	0.895	65.9	71.3	0.969	79.0	72.6	0.987
**Lymphocyte populations (general immune status)**															

T cells (CD3) (cells/µL)	869	1530	0.841	575.7	952.6	0.896	631.7	1099.1	0.839	983.2	1131.4	0.673	946.1	811.1	0.548
T helper cells (CD4) (cells/µL)	583	954	0.749	336.3	546.0	0.985	429.0	758.7	0.981	628.6	706.0	0.646	652.7	577.8	0.503
Cytotoxic T cells (CD8) (cells/µL)	242	504	0.789	173.3	334.9	0.743	158.1	266.7	0.882	268.8	352.4	0.566	219.7	194.4	0.618
B cells (CD19) (cells/µL)	99	162	0.732	111.4	75.3	0.064	101.6	165.1	0.775	273.0	373.6	0.023	132.7	73.0	0.088
NK cells (CD16/56) (cells/µL)	123	104	0.841	238.1	180.7	0.219	174.4	205.9	0.588	199.0	210.0	0.697	198.6	193.6	0.659
T cells (CD3) % of lymphocytes+A86	79	85	0.934	63.9	72.9	0.057	67.9	69.4	0.588	71.0	66.8	0.605	73.4	72.8	0.994
T helper cells (CD4) % of T cells	53	53	0.907	37.1	43.7	0.111	45.3	40.3	0.814	45.0	41.8	0.708	51.1	51.4	0.590
Cytotoxic T cells (CD8) % of T cells	22	28	0.732	21.7	24.0	0.848	17.9	23.3	0.588	19.8	20.6	0.781	16.9	17.8	0.080
Ratio CD4/CD8	2.4	1.9	0.749	2.4	2.6	0.487	3.6	2.5	0.897	2.3	2.1	0.877	3.8	3.3	0.159
B cells (CD19) % of lymphocytes	9	9	0.856	9.1	7.0	0.057	11.7	9.7	0.839	14.6	20.2	0.023	10.3	6.6	0.159
NK cells (CD16/56) % of lymphocytes	11.2	5.8	0.732	26.5	19.6	0.232	19.6	20.4	0.588	14.2	12.7	0.334	15.7	19.5	0.583
NK T-cells (CD3/56) % of lymphocytes	4.3	3.1	0.856	6.4	5.2	0.801	6.3	5.1	0.865	5.7	3.7	0.508	6.1	5.6	0.499
Double negative T cells (CD4-CD8-) % of T cells	4.7	3.6	0.907	6.2	4.9	0.920	2.1	4.0	0.986	5.5	4.6	0.697	5.3	4.2	0.318
Double positive T cells (CD4+CD8+) % of T cells	0.9	1.2	0.856	0.7	1.6	0.729	4.0	3.7	0.839	2.2	1.7	0.781	1.1	1.2	0.953
Activated T-helper cells (CD38+) % of T-helper cells	46	46	0.732	53.7	50.4	0.987	41.6	44.9	0.839	44.0	47.0	0.784	66.1	68.4	0.326
Activated T-helper cells (HLA-DR+) % of T-helper cells	11	7	0.856	15.9	15.4	0.431	10.7	16.1	0.893	15.8	10.6	0.566	8.6	9.9	0.807
Activated T helper cells (HLA-DR+CD38+) % of T helper cells	4	3	0.907	9.3	7.4	0.525	4.1	9.9	0.588	6.6	5.8	0.903	4.0	6.5	0.330
Activated cytotoxic T cells (CD8/CD38) % of cytotoxic T cells	36	49	0.856	44.4	57.4	0.050	32.4	38.0	0.588	31.8	40.2	0.546	34.9	51.3	0.000
Activated cytotoxic T cells (HLA-DR+) % of cytotoxic T cells	40	52	0.732	26.9	38.1	0.031	19.1	35.4	0.588	37.6	42.2	0.566	14.7	19.5	0.030
Activated cytotoxic (HLA-DR+CD38+) T cells % of cytotoxic T cells	13	32	0.732	17.7	29.7	0.031	10.1	23.7	0.588	18.8	26.8	0.334	7.6	14.4	0.002
**B cell subpopulations**															
Immature % of B cells	4.1	2.6	0.856	9.8	8.1	0.896	5.7	7.0	0.892	7.0	7.3	0.566	8.6	10.7	0.815
Immature (cells/µL)	4.1	4.2	0.856	10.1	5.9	0.525	5.9	11.3	0.754	6.9	16.3	0.524	12.3	6.5	0.336
Transitional (total) % of B cells	1.3	9.9	0.175	1.1	7.8	0.033	2.2	0.4	0.001	0.7	0.0	0.450	1.2	1.1	0.209
Transitional (total) (cells/µL)	1.3	16	0.024	1.1	7.4	0.032	1.9	1.1	0.001	1.2	0.0	0.681	1.3	1.5	0.030
Immature transitional B cells % of B cells	NA	NA	NA	6.7	1.4	0.848	15.5	2.2	0.775	58.0	82.5	0.524	NA	NA	NA
Immature transitional (total) (cells/µL)	NA	NA	NA	2.5	0.2	0.800	11.3	4.5	0.897	30.2	280.5	0.450	NA	NA	NA
Naive % of B cells	86.2	90	0.732	64.6	65.1	0.951	67.9	69.8	0.775	39.0	28.4	0.566	55.9	56.8	0.674

Naive (cells/µL)	85.3	145.8	0.732	66.8	52.4	0.885	70.5	103.9	0.986	100.4	58.0	0.422	60.4	41.2	0.548
Memory (total) % of B cells	4.8	3.3	0.732	15.3	17.3	0.896	7.8	14.0	0.893	32.3	30.0	0.493	22.3	18.7	0.629
Memory (total) (cells/µL)	4.8	5.3	0.988	23.3	10.7	0.743	9.5	33.8	0.839	148.7	57.4	0.023	37.5	14.9	0.318
Non-class-switched % of B cells	2	1.2	0.732	3.7	3.8	0.896	1.3	2.2	0.897	24.4	16.5	0.334	6.6	4.7	0.974
Non-class-switched (cells/µL)	2	1.9	0.936	7.3	2.5	0.987	1.4	5.4	0.775	135.9	35.6	0.018	12.8	3.7	0.606
Class-switched % of B cells	2.5	1.9	0.856	8.9	11.1	0.903	6.7	10.3	0.893	6.0	10.3	0.875	172.2	10.4	0.499
Class-switched (cells/µL)	2.5	3.1	0.907	13.5	6.7	0.896	8.5	24.5	0.839	6.4	15.1	0.334	11.5	8.2	0.345
CD21low % of B cells	NA	NA	NA	3.2	5.5	0.477	2.5	3.4	0.865	NA	NA	NA	NA	NA	NA
CD21low (cells/µL)	NA	NA	NA	1.3	0.9	0.743	3.2	8.4	0.897	NA	NA	NA	NA	NA	NA
Activated CD21low CD38low % of B cells	NA	NA	NA	2.2	3.5	0.650	1.7	2.1	0.893	NA	NA	NA	NA	NA	NA
Activated CD21low CD38low (cells/µL)	NA	NA	NA	0.9	0.6	0.885	2.4	5.1	0.775	NA	NA	NA	NA	NA	NA
Plasmablasts (CD38) % of B cells	0.2	0.2	0.856	1.4	1.2	0.987	0.8	4.5	0.588	0.9	1.4	0.831	2.0	2.2	0.590
Plasma cells (CD38) (cells/µL)	0.2	0.3	0.856	2.7	1.0	0.553	0.7	8.3	0.754	0.9	2.4	0.546	3.3	1.8	0.559
Plasma cells (CD138) % of B cells	1.5	0.4	0.880	1.5	1.0	0.903	0.7	2.1	0.955	1.5	1.2	0.833	1.7	2.2	0.227
Plasma cells (CD138) (cells/µL)	1.5	0.6	0.732	1.0	0.7	0.743	0.6	3.5	0.839	1.8	2.3	0.488	2.9	1.7	0.987
**T cell differentiation status (CD4 T cells)**															
Naive- T helper cells % of T helper cells	19.4	24.6	0.732	28.2	28.9	0.807	22.1	18.4	0.893	23.5	19.8	0.566	43.2	44.2	0.987
Naive- T-helper cells (cells/µL)	113.1	234.7	0.856	92.6	183.5	0.920	121.9	182.7	0.839	148.8	177.4	0.780	288.3	268.2	0.606
CD4 effector memory % of T helper cells	26.4	22.5	0.732	41.2	32.5	0.525	41.0	44.1	0.657	40.5	41.7	0.868	21.4	23.0	0.381
CD4 effector memory (cells/µL)	153.9	214.7	0.880	140.9	184.1	0.903	155.7	291.2	0.981	255.9	295.1	0.546	136.7	126.0	0.381
TH1 % of T-helper cells	10.3	10.7	0.907	18.7	14.3	0.837	14.7	18.7	0.897	8.2	8.9	0.895	5.4	10.1	0.759
TH1 (cells/µL)	60	102.1	0.856	73.5	61.6	0.896	65.7	100.7	0.977	55.0	60.3	0.697	29.6	50.6	0.499
TH2 % of T-helper cells	0.4	0	0.907	0.2	0.9	0.832	0.2	0.2	0.775	0.3	0.3	0.584	0.4	0.3	0.953
TH2 (cells/µL)	2.3	0	0.929	0.9	4.1	0.896	0.7	0.7	0.839	2.0	1.9	0.881	2.2	2.1	0.948
Central memory cells % of T-helper cells	41.9	40.7	0.175	18.9	20.5	0.837	24.2	23.8	0.588	21.8	20.4	0.566	19.0	20.3	0.948
Central memory cells (cells/µL)	244.3	388.3	0.732	62.8	105.4	0.937	102.9	196.0	0.839	134.9	147.3	0.781	127.9	110.7	0.326
Ratio naive/effector memory	0.7	1.1	0.732	1.0	1.2	0.650	0.9	0.5	0.897	0.6	0.7	0.896	2.3	2.9	0.496
TH17 cells % of T-helper cells	13.6	36.8	0.916	29.0	30.4	0.920	24.8	24.6	0.814	21.1	29.0	0.524	12.7	22.8	0.987
TH17 cells (cells/µL)	79	351	0.856	112.3	149.7	0.920	98.3	254.4	0.839	135.6	195.4	0.334	88.4	104.9	0.207
**T cell differentiation status (CD8 T cells)**															
Naive CD8 T cells (CD45RA+CCR7+) % of cytotoxic T cells	23.4	19.7	0.856	19.8	18.2	0.743	18.4	12.8	0.637	7.3	7.8	0.931	29.3	32.6	0.739
Naive CD8 T cells (CD45RA+CCR7+) (cells/µL)	56.6	99.3	0.732	28.9	40.5	0.848	21.8	34.6	0.835	19.6	24.6	0.984	65.3	59.3	0.815

Effector memory (CD45RA-CCR7-) % of cytotoxic T cells	39.7	38.3	0.732	31.2	30.2	0.525	34.9	39.4	0.977	38.3	43.0	0.334	29.4	30.6	0.948
Effector memory (CD45RA-CCR7-) (cells/µL)	96.1	193	0.561	62.5	72.3	0.987	58.4	116.0	0.991	103.3	142.5	0.334	60.2	54.7	0.763
Activated effector memory (CCR5+) % of cytotoxic T cells	2.4	0.7	0.856	1.6	1.0	0.937	0.4	1.3	0.893	0.3	1.8	0.546	2.7	1.0	0.967
Activated effector memory (CCR5+) (cells/µL)	5.8	3.5	0.749	2.8	1.1	0.987	0.9	2.2	0.839	0.9	6.3	0.566	2.6	2.2	0.987
Activated cytolytic effector cell (CCR5+) % of cytotoxic T cells	0.6	0.1	0.856	0.2	0.2	0.729	0.2	0.8	0.897	0.1	0.4	0.857	0.4	0.3	0.618
Activated cytolytic effector cell (CCR5+) (cells/µL)	1.5	0.5	0.945	0.3	1.4	0.896	0.4	1.0	0.839	0.4	1.5	0.697	0.5	0.7	0.499
CD8 effector cells (CD45RA+CCR7-) % of cytotoxic T cells	17.9	27.7	0.933	33.0	33.2	0.221	26.3	23.6	0.897	34.1	31.2	0.458	24.5	23.7	0.987
CD8 effector cells (CD45RA+CCR7-) (cells/µL)	43.3	139.6	0.789	76.3	166.2	0.285	49.2	66.2	0.893	92.6	125.2	0.697	54.6	52.4	0.967
Central memory cells % of cytotoxic T cells	5.3	4.8	0.789	3.8	3.7	0.732	7.9	5.8	0.865	5.1	6.0	0.697	5.5	5.3	0.209
Central memory cells (cells/µL)	12.8	24.2	0.732	7.2	9.1	0.985	10.0	14.7	0.945	14.1	20.5	0.546	14.4	12.2	0.309
**Regulatory T cells**															
Regulatory T cells % of T cells	5.2	5.1	0.732	6.3	7.6	0.896	9.0	6.2	0.775	7.3	6.4	0.896	5.7	6.6	0.739
Regulatory T cells (cells/µL)	45.2	78	0.933	33.7	56.3	0.989	45.7	55.2	0.775	72.1	72.9	0.697	58.3	54.8	0.313
Interleukin 2 receptor % of T cells	49.4	43.3	0.933	41.0	44.8	0.285	49.1	46.6	0.588	48.5	48.8	0.524	43.0	48.3	0.974
Naive- T helper cells % of T helper cells	19.4	24.6	0.732	28.2	28.9	0.807	22.1	18.4	0.893	23.5	19.8	0.566	43.2	44.2	0.987
Naive- T-helper cells (cells/µL)	113.1	234.7	0.856	92.6	183.5	0.920	121.9	182.7	0.839	148.8	177.4	0.780	288.3	268.2	0.606
CD4 effector memory % of T helper cells	26.4	22.5	0.732	41.2	32.5	0.525	41.0	44.1	0.657	40.5	41.7	0.868	21.4	23.0	0.381
CD4 effector memory (cells/µL)	153.9	214.7	0.880	140.9	184.1	0.903	155.7	291.2	0.981	255.9	295.1	0.546	136.7	126.0	0.381
TH1 % of T-helper cells	10.3	10.7	0.907	18.7	14.3	0.837	14.7	18.7	0.897	8.2	8.9	0.895	5.4	10.1	0.759
TH1 (cells/µL)	60	102.1	0.856	73.5	61.6	0.896	65.7	100.7	0.977	55.0	60.3	0.697	29.6	50.6	0.499
TH2 % of T-helper cells	0.4	0	0.907	0.2	0.9	0.832	0.2	0.2	0.775	0.3	0.3	0.584	0.4	0.3	0.953
TH2 (cells/µL)	2.3	0	0.929	0.9	4.1	0.896	0.7	0.7	0.839	2.0	1.9	0.881	2.2	2.1	0.948
Central memory cells % of T-helper cells	41.9	40.7	0.175	18.9	20.5	0.837	24.2	23.8	0.588	21.8	20.4	0.566	19.0	20.3	0.948
Central memory cells (cells/µL)	244.3	388.3	0.732	62.8	105.4	0.937	102.9	196.0	0.839	134.9	147.3	0.781	127.9	110.7	0.326
Ratio naive/effector memory	0.7	1.1	0.732	1.0	1.2	0.650	0.9	0.5	0.897	0.6	0.7	0.896	2.3	2.9	0.496
TH17 cells % of T-helper cells	13.6	36.8	0.916	29.0	30.4	0.920	24.8	24.6	0.814	21.1	29.0	0.524	12.7	22.8	0.987
TH17 cells (cells/µL)	79	351	0.856	112.3	149.7	0.920	98.3	254.4	0.839	135.6	195.4	0.334	88.4	104.9	0.207
**T cell differentiation status (CD8 T cells)**															
Naive CD8 T cells (CD45RA+CCR7+) % of cytotoxic T cells	23.4	19.7	0.856	19.8	18.2	0.743	18.4	12.8	0.637	7.3	7.8	0.931	29.3	32.6	0.739

Naive CD8 T cells (CD45RA+CCR7+) (cells/µL)	56.6	99.3	0.732	28.9	40.5	0.848	21.8	34.6	0.835	19.6	24.6	0.984	65.3	59.3	0.815
Effector memory (CD45RA-CCR7-) % of cytotoxic T cells	39.7	38.3	0.732	31.2	30.2	0.525	34.9	39.4	0.977	38.3	43.0	0.334	29.4	30.6	0.948
Effector memory (CD45RA-CCR7-) (cells/µL)	96.1	193	0.561	62.5	72.3	0.987	58.4	116.0	0.991	103.3	142.5	0.334	60.2	54.7	0.763
**NK cell subpopulations and activation**															
Immature NK cells I (CD56-CD16-) % of NK cells	1.7	0.9	0.732	0.1	0.2	0.243	0.3	0.5	0.882	0.4	0.2	0.857	0.4	0.4	0.953
Immature NK cells II (CD56+CD16-) % of NK cells	14.7	13.9	0.122	3.3	4.4	0.057	3.6	4.2	0.588	3.2	4.3	0.508	4.5	5.4	0.159
Mature NK cells (CD56+Cd16+) % of NK cells	69.8	57.2	0.732	90.8	81.8	0.158	83.5	85.5	0.991	77.9	76.3	0.392	84.6	84.5	0.949
CD56bright Nk cells (regulatory effects) % of NK cells	1.3	7.6	0.130	2.8	4.4	0.729	2.9	3.7	0.588	3.1	6.0	0.546	2.9	3.5	0.548
CD16+CD56- Nk (cytolytic activity) % of NK cells	7.1	21.3	0.856	4.2	11.2	0.920	10.5	7.7	0.775	15.9	15.3	0.718	8.3	7.6	0.059
Activated CD94/NKG2D complex % of NK cells	8.9	28.8	0.856	36.0	39.4	0.985	31.7	27.9	0.853	32.4	41.4	0.833	42.0	41.2	0.629
**NK cells and activation level**															
Activation signal Nkp30 % of NK cells	77.8	63.1	0.732	81.1	73.7	0.800	76.8	76.3	0.897	62.8	57.1	0.566	61.6	79.4	0.930
Activation signal Nkp46 % of NK cells	44.6	46.2	0.856	62.7	57.9	0.985	46.5	53.2	0.865	44.5	42.3	0.711	57.1	64.0	0.618
Activation signal Nkp44 (cytokine. Akt.) % of NK cells	0.1	0.7	0.907	0.5	0.6	0.920	5.1	1.2	0.588	2.1	0.6	0.780	0.6	0.8	0.953
Activation signal CD57 % of NK cells	56.6	43.3	0.732	47.7	43.2	0.650	44.1	44.0	0.977	42.9	39.4	0.646	55.0	55.6	0.604
**Phagocytosis ops. E. coli***															
Phagocytosis rate granulocytes % of granulocytes.	99.5	99.6	0.907	98.3	99.1	0.920	97.7	98.3	0.954	98.5	98.2	0.976	99.1	98.9	0.590
Phagocytosis rate monocytes % of monocytes	98.3	97.5	0.856	89.9	97.7	0.717	94.0	90.8	0.991	97.3	90.6	0.857	96.2	94.2	0.987
Phagocytosis intensity granulocytes	54.2	32.3	0.732	60.7	64.1	0.920	48.0	55.9	0.897	72.8	62.4	0.896	88.1	64.6	0.499
Phagocytosis intensity monocytes	53.9	39.8	0.732	55.4	64.4	0.920	38.9	48.5	0.899	61.9	46.6	0.646	83.6	70.9	0.953
**Oxidative burst ops. E. coli***															
oxid. Burst Granulocytes positive Contr. % of granulocytes	97.6	86.6	0.561	96.1	96.9	0.717	88.5	91.7	0.769	93.2	99.2	0.334	99.6	98.3	0.948
The proportion of oxid. Granulocytes % of granulocytes	99.6	98.3	0.749	98.3	98.6	0.896	96.3	98.1	0.991	98.4	99.1	0.334	99.1	98.9	0.629
oxid. Burst Granulocytes	12.36	6.74	0.732	21.0	18.4	0.987	14.2	19.7	0.588	17.4	29.6	0.126	17.8	28.3	0.953
Proportion oxid. Monocytes % of monocytes	97.3	91.0	0.749	92.1	96.0	0.946	94.0	86.9	0.775	95.1	98.1	0.565	92.4	91.3	0.499
Burst intensity monocytes	3.36	3.11	0.732	7.3	5.7	0.174	5.5	6.8	0.865	6.2	6.5	0.546	6.2	6.5	0.618
**Chemotaxis of granulocytes***															
Number of migrated granulocytes	9.68	4.56	0.732	15.8	13.8	0.903	20.4	13.5	0.588	8.8	15.4	0.984	20.0	12.0	0.115

A number of migrated granulocytes neg. control	6.84	2.0	0.933	1.4	4.5	0.057	1.2	3.3	0.839	3.5	9.1	0.023	6.2	5.5	0.642
The proportion of active. Granulocytes (L-selectin) % of granulocytes.	99.8	99.8	0.934	99.9	99.1	0.431	99.5	92.6	0.758	99.8	96.2	0.018	99.5	99.6	0.930
The proportion of active. Granulocytes (L-selectin) neg. contr % of granulocytes	14.8	20.6	0.732	11.3	13.1	0.985	6.4	20.0	0.993	25.1	17.3	0.646	14.1	19.3	0.313
CD21lowCD38low (cells/µL)	221.0	96.0	0.856	381.6	310.4	0.525	263.4	444.4	0.893	458.2	882.2	0.533	541.1	334.3	0.313
CD21lowCD38low %	5.0	2.1	0.856	11.4	10.2	0.717	6.0	7.6	0.897	13.8	13.7	0.697	13.5	12.6	0.987
CD21low (cells/µL)	108.0	27.0	0.732	172.3	97.3	0.221	107.6	187.7	0.839	278.2	533.4	0.524	379.4	192.9	0.381
CD21low %	2.5	0.6	0.732	4.7	3.7	0.937	2.4	3.8	0.775	8.4	8.3	0.566	8.2	7.0	0.815
Activated CD21lowCD38low (cells/µL)	74.0	20.0	0.856	92.7	54.0	0.221	76.0	112.7	0.865	233.4	454.2	0.458	273.4	116.0	0.318
Activated CD21lowCD38low %	1.7	0.4	0.732	3.1	2.4	0.975	1.6	2.2	0.894	7.0	7.1	0.781	5.9	4.3	0.967

* = Granulocytes phagocytosis, oxidative burst, and chemotaxis tests were performed using Phagotest-Kit, Phagoburst-Kit, and Migratest-Kit, respectively, from Celonic, Heidelberg, Germany following standard protocols. In brief, phagocytosis: heparinized whole blood was split into two samples of 100 µl (negative control and E. coli stimulated) and pre-cooled on ice. After 10 minutes each sample was incubated with 20 µl E. coli (FITC-pre-stained) either on ice (negative control) or at 37oC (stimulated) for 10 minutes. After that, 100 µl of quenching solution were washed in each sample. Samples were washed with 3 ml washing buffer by centrifugation (500 x g, 5 minutes, 4oC) twice. Then, lysing buffer was used to remove erythrocytes. Finally, for DNA-staining 200 µl of propidiumjodid solution was added to each sample for 10 minutes on ice. All components were used. Oxidative Burst: heparinized whole blood was split into three samples of 100 µl (negative and positive control and E.coli stimulated) and pre-cooled on ice for 10 minutes. For stimulation oxidative burst activity, 20 µl of either washing buffer (negative control), E.coli (E. coli stimulated), or PMA solution (positive control) were given in the samples and incubated for 10 minutes at 37oC. Then, lysing buffer was used to remove erythrocytes. After that, cells were centrifugated and washed with 3 ml washing buffer (500 x g, 5 minutes, 4 oC). Finally, for DNA-staining 200 µl of propidiumjodid solution was added to each sample for 10 minutes on ice. Chemotaxis: granulocytes were isolated by gently overlaying of 1 ml heparinized whole blood on a separation medium (Reagent A). After 40 minutes at room temperature, leucocytes were enriched in the upper phase. Then, two wells of a 24-well cell culture plate were either filled with 350 µl incubation buffer (negative control) or fMLP stimulation buffer (stimulated sample). A cell culture mesh was inserted into the wells and filled with 100 µl of leucocyte-enriched plasma. After 30 minutes incubation at 37 °C, cell culture inserts (mesh) were removed and the cell suspension from the bottom of the wells was transferred into a new reagent tube on ice. Additionally, 20 µl of the suspension from the cell culture insert (mesh) from negative control was removed and incubated with 180 µl incubation buffer on ice. That sample used stimulation control (CD62-Ligand measurement). Furthermore, each sample was incubated with 20 µl of antibody cocktails (Reagent D) for 10 minutes on ice. Finally, for DNA staining 20 µl of propidiumjodid solution was added to each sample for 5 minutes in the dark.

1.1 Supplementary Figures

I. B cell gating strategy

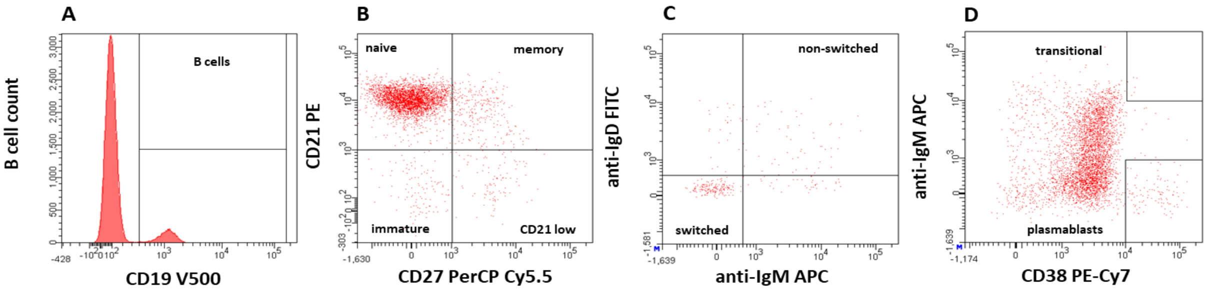

II. T cell gating strategy

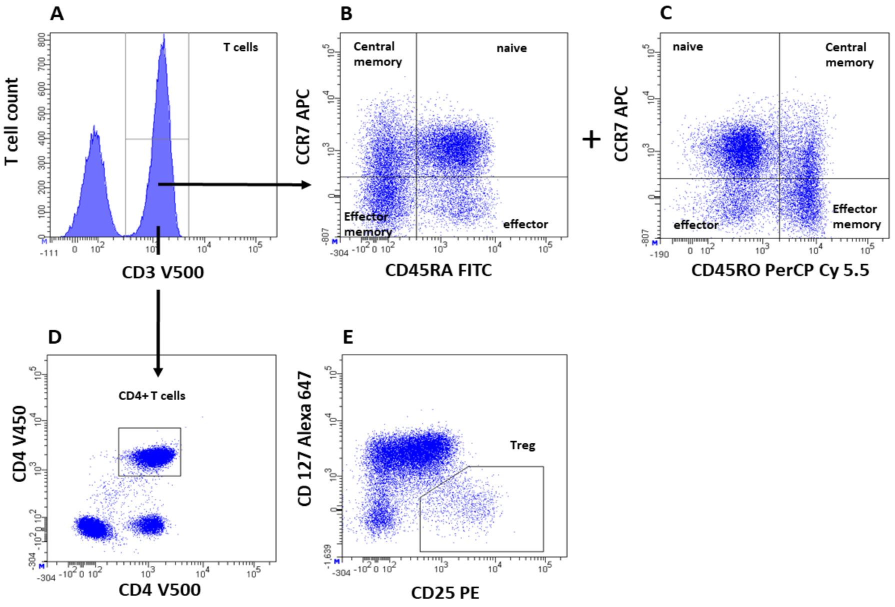

III. NK cell gating

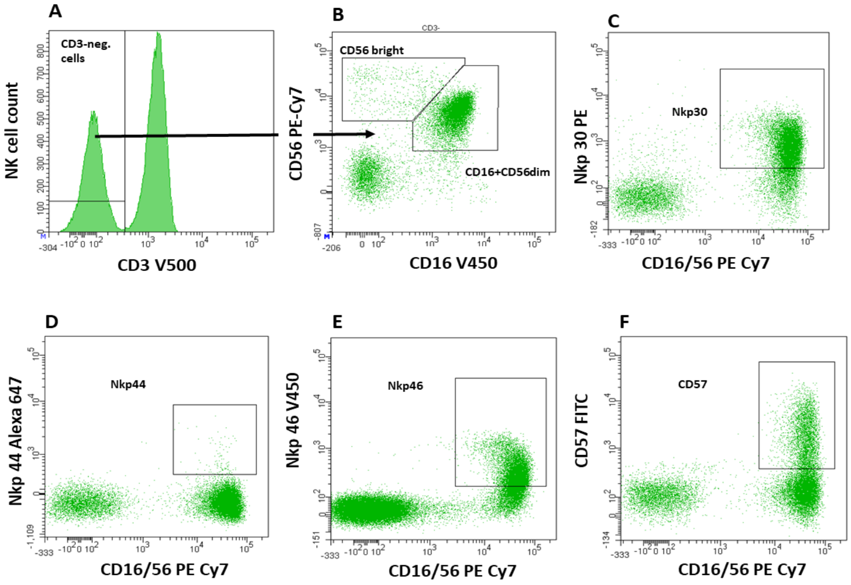

Supplementary Figure S1. Getting strategy of B cell (I). T cell (II) and NK cell (III) subpopulations. (I) CD19 staining was used to separate B cells (IA) from lymphocytes. For B cell subset analysis, the CD21 versus CD27 were used to discriminate between naive (CD21+CD27-). memory (CD21+CD27+). CD21low (CD27+CD21-) and immature (CD21/CD27 double negative) (IB). From the memory cell population (IB) class-switched and non-switched B cells were detected by surface staining with IgD and IgM (IC). Additionally, transitional B cells showed a high expression of CD38 and IgM in contrast to plasmablasts which were CD38 high but IgM negative (ID). (II) CD3 staining was used to separate T cells (IIA) from lymphocytes. For T cell subset analysis, the combination of CCR7, CD45RA, and CD45RO were used to discriminate between naive (CD45RA+CD45RO-CCR7+), effector memory (CD45RA-CD45RO+CCR7-), central memory (CD45RA-CD45RO+CCR7+), and effector cells (CD45RA+CD45RO-CCR7-) (II B. IIC). Additionally, from CD3+CD4+ T helper cell population (IID), regulatory T cells (Treg) were detected as CD25+CD127 low cells (IIE). (III) For NK-cells subsets analysis, T cells were excluded by gating on CD3 negative cells (IIIA). Based on CD3 negative cells, CD16 and CD56 markers were used to discriminate between mature NK cells (CD16+CD56dim) and CD56 bright NK cells (IIIB). In the next step, the expression of NK-cell activation markers on mature NK cells was analyzed: Nkp30 (IIIC). Nkp44 (IIID). Nkp46 (IIIE). and CD57 (IIIF).

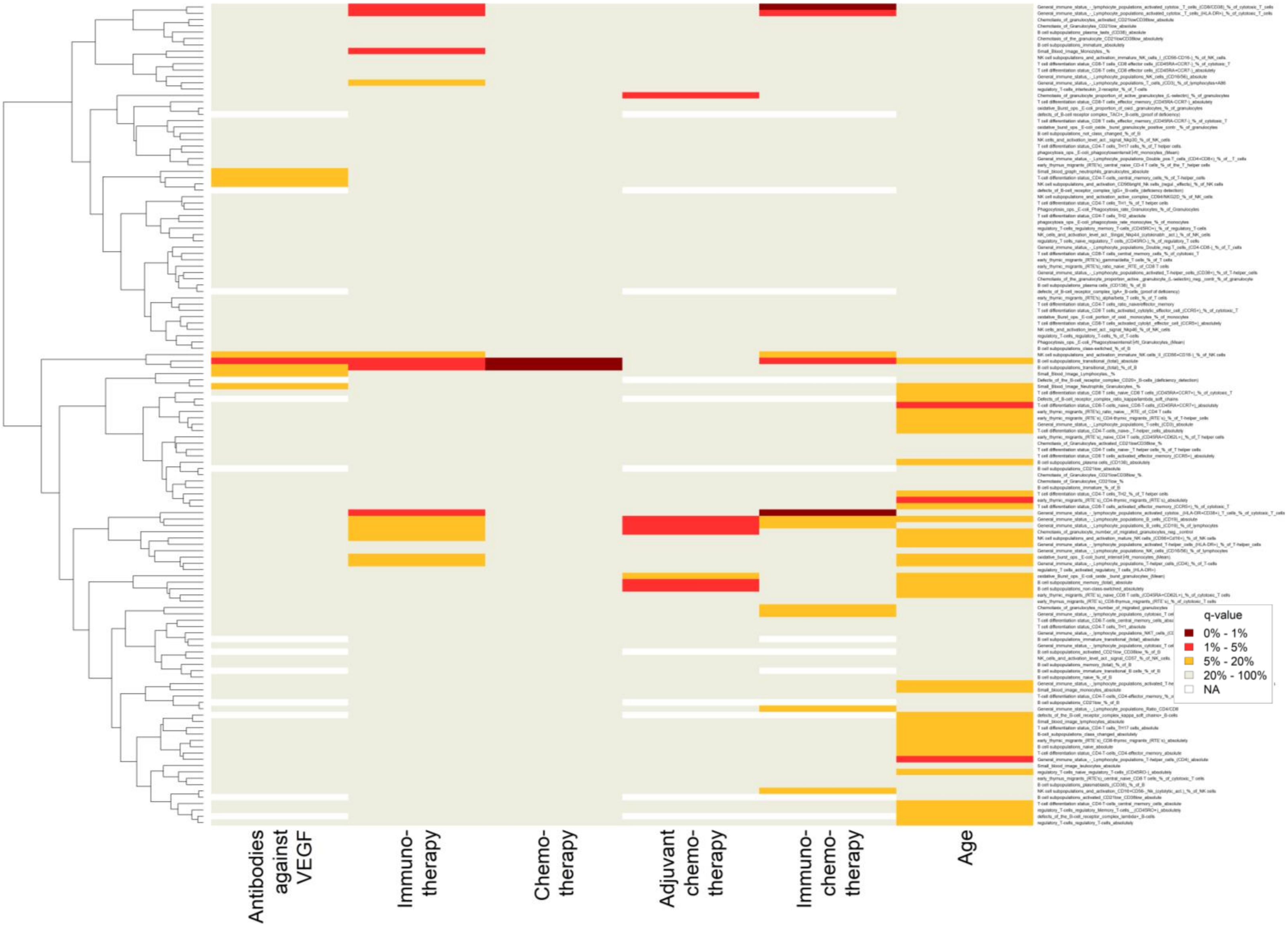

Supplementary Figure S2. Heatmap showing changes in lymphocyte subpopulations after treatment with different anti-cancer therapies and aging. Statistically significant changes in lymphocyte subpopulations are indicated by dark red and red color boxes (q = 0 - 1% and 1 - 5%, respectively). The dendrogram shows the group of lymphocyte subpopulations according to hierarchical cluster results based on the statistical association strength.
